# On the Aborigines of New Holland and Van Dieman's Land

**Published:** 1823-09

**Authors:** 


**Art. II.**—*On the Aborigines of New Holland and Van Dieman's Land.* By an Army Medical Officer.*

I have endeavoured to gain, respecting the native tribes, their condition, manners, and customs, all the information possessed by the colonists, and that such among themselves as speak English are capable of affording; but my opportunities have been few, and the time I have been among them too short to enable me to do more than offer a few hints relative to those in the immediate neighbourhood of Port Jackson.

The history of these people is a subject which has been little attended to by the English settlers; and which is, from their nature and habits, very difficult of investigation. The whole of the aborigines of this great country, and the neighbouring islands, as far as they have been examined by Europeans, occupy a low place in the scale of the human species; but, as far as I am able to judge, they are not quite so abject, or so incapable of receiving instruction, as they are represented. Of these within and about the British colony, the language and manners are fast decaying; and in a few years it may be difficult to find a tribe to convey an idea of what they have originally been, or even are now.

* The source whence we have derived this communication leads us to place confidence in its accuracy.—Editors.

The natives of Van Dieman's Land I have not seen; but, by all the accounts I have heard, they are still lower in the scale of being than those of New South Wales. From the unsociable disposition of the males, and the hatred they bear the colonists, little is known respecting them; and that little is most probably much disfigured in the meagre accounts given to the public, in all which they are stated to be very little superior to wild beasts. They are rapidly diminishing in numbers; and, as colonization advances, they will still further decrease. In New South Wales they are not decreasing in the same ratio, as they have not been molested to the same extent by the white people; but they are not now so numerous as they were on the first establishment of the colony, and, from the habits of drunkenness and other vices gaining ground among them, it is not likely that they will preserve their present numbers long. In both countries they have made less progress towards civilization than any other people possessing a temperate climate, and a soil capable of being made productive. They have neither houses nor clothing; nor does it appear that they ever had the least knowledge of the cultivation of the earth, or of raising any kind of food, but what was spontaneously produced to their hand. In a few instances they have trained the native dog, and made him subservient to their use in the chase of the kangaroo and other animals; and that appears to be the only animal they have attempted to tame. Their only shelter from the weather is the hollow of trees or rocks. The construction of the simplest hut, further than sticking a few pieces of bark on end, and tying them together, they never attempt, and seems, in fact, to be above their reach. Except now and then an occasional slip of kangaroo or opossum skin hung over their shoulders, they have nothing whatever to cover their bodies in the coldest weather of the climate; and at all seasons they walk about without seeming to suffer the slightest inconvenience.

They have certainly not gained much by their intercourse with civilized people. In the neighbourhood of towns they have learnt to value clothing, and endeavour to obtain it; but they have not as yet practised one of the arts of civilised life. Many of the worst habits of the Europeans they have acquired, and practise assiduously. All those who mix with the colonists, men and women, are addicted to drinking spirits to excess; they are also fond of tobacco; and, whenever they have an opportunity, they beg with great clamour and obstinacy, and even prescribe the actual sum they expect to receive, which is always proportioned to the appearance, or their knowledge of the person solicited. I am not, however, aware that they are often guilty of theft; and instances of their honesty, which would do credit to any people, have come within my own knowledge, and that when they could have taken property without a chance of detection."

The colour of these people is a dark brown, or very nearly black. Their features are decidedly African: they have flat noses, large nostrils, eyes sunk, the mouth very wide, and both lips even of greater thickness than in most natives of Africa. The colour of the hair is in some jet black; in others it resembles that of the skin. It is harsh to the feel; in some perfectly straight, and in others twisted into ropes, which hang about the head exactly in the form of a mop. This, if not natural, is increased by art, and reckoned a beauty. The men have strong matted beards, which in the woods they allow to grow, but in the colony they keep shaved or close cropped, in imitation of the whites. In all that I have had an opportunity of examining, the head is narrow, compressed laterally, with the cheek-bones projecting forwards. The lower jaw is strong, and projecting forward; the cranium thick and heavy. They are in general of a low stature, thin and light made, with small limbs, but not otherwise ill-formed, as has been represented. A few men may be met with among them above the middle size, with broad chests, and as well-turned limbs as any European. These instances are not common, but they may be seen every day among the tribes who come from the distant parts of the colony to visit the towns. The features of the women are not unpleasant, though the negro countenance is strongly marked. They are, almost without exception, low in size, with small bones, and very thin: when old, they are extremely wrinkled and ugly. The men bore a hole through the septum nasi, through which they pass a piece of reed or bone. This ornament is only worn on occasions of ceremony, and it gives them a most disgusting look. Those in the habit of mixing with the colonists seldom wear any thing in the nose, though they all have the septum pierced. The women ornament their hair with strings of kangaroo teeth; and both sexes, when mourning for their relatives, paint their forehead and cheeks with streaks of white clay.

They possess an acuteness of the senses which is particularly remarkable: even those of the most vitiated habits in and about Sydney, have the power of hearing, seeing, and smelling, to a degree quite incredible to Europeans. Sounds inaudible to our ears make an impression on their organs, and they can trace in the woods, and on the bark of trees, impressions left by men and animals, where we can perceive nothing but grass and leaves. This faculty they have in such perfection, that they are employed in tracing run-aways and bush-rangers; and they very seldom fail in apprehending them. They follow them through the closest woods and most intricate bye-paths; for days; and they can readily tell, from a slight impression of the foot on the grass or fallen leaves, whether the parties are blacks or whites, friends or foes.

Almost all these people possess quick conceptions, and their powers of imitation are surprising. There is hardly a man of the least note in the colony whose actions and manners are not represented by the most lazy and indolent of these fellows who frequent our streets, in a way that cannot be mistaken by any one, and in the most artless and natural manner, far more correct and resembling the original than the best efforts of our ablest professors of the art of mimicry. But, with all this readiness and apparent talent, they have no reflection, judgment, or foresight. They have never been in the slightest degree cultivators; and their mechanical talents are of the lowest order, as is shown by their implements for war and the chase, which are of the rudest manufacture. Their canoes consist of a piece of the bark of the box-tree (of the colonists,) tied at each end, in which they never venture any distance from the shore.

They have no civil government extending beyond a family, or at most a small tribe; and I have not been able to learn that they have any religious superstition, formed into any kind of system, like the natives of the South-Sea Islands. They have a vague belief in a Deity and a future state; but some of them, who spoke English with fluency, and expressed themselves with precision on ordinary topics, were not able to give me any consistent account of their religion, or their superstitions; but that they believe in ghosts is evident. They have no particular person set apart to exercise religious offices; or, indeed, any office except that of chief, which among some tribes is hereditary, and in others is conferred on the man of greatest personal courage and conduct in war. Their obedience to these chiefs appears to be entirely voluntary; and the only advantage I have been enabled to learn which he derives from his dignity is that, in dividing the spoils of a hunting or fishing day, he has the first and choicest share. As they have no settled abode, they make no provision for the future; the only care they have is to provide the meal of the day that is passing.

They have, notwithstanding their migratory life, an idea of possessing a property in the land, not only as whole tribes or families, but individuals say that particular districts are theirs by hereditary right, and will often pretend to dispose of them to the present occupiers. The tribes, though seldom stationary, never travel far, and they do not appear to interfere with each other's range. Thus we have the Sydney, the Broken Bay, the Botany Bay, Kissing Point, &c. tribes, all of whom meet and mix at Sydney; but, when they leave the town, they each retire to their own district, and consider the woods and rocks of it as their native home.

Every man wants a front tooth. This is extracted at puberty, at a general meeting of several tribes; but for what purpose is not exactly ascertained. It is probable that it is a mere symbol of the youth having attained manhood, and being entered on the list of fighting men. Both men and women have deep scars on various parts of the body; but chiefly about the shoulders there appears to be a kind of rude tattooing, done by way of ornament; or perhaps, as I have been informed, they are intended to distinguish the people of one tribe from another, or to know their children, should they be lost.

By much the greater part of their food is derived from the animal kingdom: kangaroos, opossums, guanas, and indeed every species of animal they can catch. They almost always spear them, at the use of which weapon they evince much dexterity, and seldom miss an animal at the distance of forty or fifty yards, though in full speed. Some kinds of insects, and in particular grubs and larvæ of a species of moth, which inhabits the bark of the mimosa, or green wattle, they esteem a rich and favourite article of food.

The tribes near the coast and on the bays live a good deal on shell-fish, oysters, &c. which are very abundant. They are good fishers, and some have overcome their native indolence so far as to bring fish to market for sale; the only way in which I have ever known them to exercise industry. In catching fish, they now make use of hooks and lines; but their own mode is by a spear of a peculiar light construction, at the use of which they are very expert. The fishing is chiefly left to the care of the women; and those engaged in it have the first joint of the little finger of the left hand taken off. This is done in early life, by means of a ligature. Whether they conceive this mutilation fits them more for the business of fishing, or that it acts as a charm, I do not know. I have often made the inquiry, and the answer always was, that, without the finger being taken off, the women would not catch many fish.

Their only mode of cooking is by broiling or roasting, or rather scorching, the meat on a hasty fire of green wood; and, when they catch an animal of any kind, they throw it on entire, without cleaning or cutting, and eat the parts of it in succession as they become cooked to their taste.

There are no native fruits in the country near the coast, nor in any part of it, as far as I am able to learn. The native cherry, with the stone on the outside, ~~the fruit of the *exocarpus cupressiformis*~~, hardly forms an exception. The only vegetables they make use of are the roots of the different kinds of ferns, with which the woods abound. They undergo no other preparation than pounding between two stones, and, by the addition of a little water, they form a kind of paste, which they eat with their animal food. They also use a sort of bulbous root, like a wild yam, and the gums from different trees, but chiefly that from the mimosa, (green wattle of the colonists.) These articles, however, they seldom resort to in latter days, but when the hunting or fishing fails, or when they are at too great a distance from settlements, or the colonist refuses them bread.

They have few diseases, but such as arise among those near towns from intemperance and neglect of themselves: like the white inhabitants, they are subject to bowel complaints, and they consider the gum of the mimosa a sovereign remedy in these affections. I have not been able to ascertain whether they obtained the knowledge of any medicinal virtues this gum possesses from the colonists, or the colonists from them; but they both make use of it, and consider it of great service in dysentery. The root of the fern they esteem diuretic, and they use it in gonorrhœa and other affections of the urinary organs. This, I believe, is the extent of their medical skill: their native doctors never venture further. The chief part of their art is confined to charms, which consist in repeating some set words over the patient; but of the meaning of them, or their supposed efficacy, I could gain no other information than that they deem it right always to undergo such a ceremony when they are very ill. The head man of the tribe is very generally doctor. They are fond of applying to the European medical men for advice, but they can seldom be got to take medicine: A glass of Bengal rum, or, in the colonial phraseology, "Bull," is their great panacea; and to it they resort whenever they can obtain it. In this they implicitly follow the practice of their civilised brethren, among whom Bengal rum forms the only enjoyment when well, and the only medicine when ill.

The surgery of the native tribes is equally simple with their medical practice, but more efficacious. When bit by snakes, which is with them a frequent occurrence, they make a ligature above the wound, scarify it with a shell or any sharp instrument they possess, and then suck it for a considerable time. The women exercise this branch of the art, and when they are at hand, the colonists, who meet with accidents of this kind, always apply to them; and, if this simple operation is performed soon after the injury is inflicted, the deleterious effects of the poison are generally prevented.

In the quarrels of one tribe with another, and sometimes in squabbles of a more domestic kind, they frequently receive very severe wounds, inflicted both by their clubs, or *waddies*, and their spears; yet it rarely happens that any serious injury follows. I have seen deep and ragged wounds of the scalp, penetrating to the bone, heal in a wonderfully short time, with hardly any attention on the part of the patient, and no confinement; and fractures of the upper extremities do well, and are cured without any deformity and without any application, and with just as little care on their part, or attention on that of their friends.

These frays most generally take place about their women. Marriage is agreed on between the parents when the children are very young, and they live with their families till they are by them deemed fit to come together; after which the woman always remains with her husband. If a girl arrives at the age of ten or twelve years without being given away, or if any accident has happened to her betrothed, any man who fancies her seizes an opportunity of carrying her off from her parents, and, if she is unwilling to go with him, he drags her by force, and beats her with his club (waddie) till she is insensible, or till she follows him wherever he chooses: after this the parents never interfere. But, if she happens to be the favourite of another, he attempts her rescue from his rival. If she is willing to go with him, their claim is settled by single combat. A regular challenge is given: the claimants decide the question before the whole tribe, according to certain laws regularly established, and which are attended to with more form and more honour than could be expected from them.

Upon all occasions in which an individual has cause of complaint against one of another tribe, he sends him notice to fight him at a particular time and place, giving him the choice of weapons,—either the death or hunting spear; but mentioning which of these he himself prefers. If the challenge is accepted, the parties meet, accompanied by their respective tribes, and others friendly to them. The challenging parties paint their faces and other parts of their bodies with streaks of red ochre, and as many of their friends as choose to fight in their cause do the same. Every one who thus paints himself is under the necessity of fighting, if challenged by any one of the opposite side. The first business of the day is to decide the quarrel of the party that led to the meeting, and their friends engage afterwards, as they may be disposed to show their courage, and do honour to those they have attended to the field.

They have generally but one wife, but it does not appear that they have any law to prevent their having more. ~~I have seen some of them attended by two women they called their *gins* (wife).~~ Codgie, chief of a tribe on the Nepean, has two wives, who always go about with him: I asked why he had more wives than the rest of his people? but, though he speaks English very well, he would give me no other answer to several questions I put to him, than that it was right he should have two.

Females in a state of nature think little of the evils attendant on parturition, so formidable in civilised life. Among them it is easy, and the day after the woman walks about her usual business. They wrap the child in the soft bark of the tea-tree, which is a substance admirably calculated to preserve them from the air, and defend them from injury. How long they continue to give them this covering, I do not know, but I should think not above a few days, as they go about with young infants in their arms in a state of perfect nudity; and, as soon as it is able to support itself, it sits on the mother's shoulder, holding by her hair.

The men never carry any thing but their arms, consisting of their spears, the waddie or club, and the *boomering*, a sort of wooden sword, much curved, which they throw with great force and surprising precision. In their combats they use a clumsy oblong shield, made of the very hard and heavy wood of the iron bark-tree; but they only carry these about with them when they are going to battle. The women carry whatever moveables they possess, but they are always very trifling, and never exceed the provisions for the day. They sling them in a net made of the fibres of the stringy bark-tree: these they plait together, and suspend them from the shoulders by a string of the same material, which lets the bag hang low on the loins. A kind of excrescence growing on the bark of the tea-tree, which they scoop out into a rude cup to hold water, and a piece of bark tied at both ends in the form of a canoe, are the only utensils I have ever seen with them.

As far as the experience of the European settlers as yet goes, the inference must be drawn that the natives of New South Wales will never be a civilised or industrious community. They have been intimately mixing with the colonists above thirty years, and many efforts have been made, and still are making, to induce them to settle, and avail themselves of the arts of civil life; but they cannot be fixed, nor is it possible, by any kindness or benefits, to attach them to one place. They have been brought up from infancy in our schools, and yet at maturity they betook themselves to their native woods, and showed at once the savage instinct which their civilised education had only smothered. I do not know that there is a single instance of one brought up among us having become a settler, or having in any way acquired property, or turned his education and acquirements to any useful purpose.

Notwithstanding Blumenbach's making a distinction between the natives of the east and west coast, I am of opinion that all of the aborigines of Australasia are of Ethiopian origin. As far as I have been able to trace, they possess not only the exterior, but all the degenerate qualities, of the African negro. The only perceptible difference is in their straight hair; every thing else is the same. It has been said that tribes of natives with woolly hair have been seen on the north-west coast of the island: this is, however, without foundation. Captain King, who saw more of the coast than any other European, never met any such; nor am I aware of any person who has observed, in that respect, the least difference among them throughout the whole country. I would, however, lay no stress on a diversity of hair as showing a difference of origin. The natives of Van Dieman's Land have woolly hair, but there appears to be no other distinction between them: their colour, genius, and habits, are the same with those of this colony; but they are more miserable, from the effects of climate and the difficulty of procuring subsistence; and they are less tractable, and more shy of strangers, perhaps more from the inhuman treatment they have experienced than from their natural disposition. The difference in Africa between the neighbouring tribes of the Hottentots and Caffres (both Ethiopians,) is infinitely greater: the former being short, slight, ill-formed, and of a yellow hue, with flat noses, and high cheek-bones; whilst the latter are tall, finely formed, and of a deep-brown colour, with the high forehead and prominent nose of the European.

Of the tribes in the vicinity of the coast, (who are, of course, best known,) very few are found who have attained old age. There is hardly a person to be met with who remembers Captain Cook being at Botany Bay in 1770, and not many even who recollect the founding of the colony in 1788. None of them can tell their age exactly; but, from the appearance of many who have been well known to the colonists for years, I should suppose fifty to be old age, and sixty to constitute extreme old age, and a period to which very few attain.

The most philosophical division of the varieties of the human species is certainly that of Professor Blumenbach, into the Caucasian, Mangolian, the Ethiopian, the Malayan, and the American. I have already stated my belief that the natives of Australasia are of African origin, notwithstanding their straight hair; and I shall now proceed to adduce such farther testimony in support of this opinion as I have been able to derive from reading and conversation with intelligent men.

Dr. Pritchard, in his inaugural disputation on the varieties of the human species, says, "The islands of the Indian Sea, as well as those of the Pacific, contain two races of men, differing in many respects. One of these approaches, and in some instances equals, the blackness of the negro; the hair is curled and woolly, the body slender, the stature short, the disposition

[Feb. 10, 1824. Sydney Gazette
Times June 21, p. 3-c. 1

barbarous and cruel: the other is more like the Indians of the continent, has a fairer skin, larger limbs and stature, better porportioned, and exhibit some marks of humanity and civilization. According to Foster, the former, who are aborigines, have occupied mountainous parts of many islands, leaving the coast and plains to the more recent colonists. They occupy the highest parts of the Moluccas, the Phillipines, Formosa, Borneo, all New Guinea, New Britain, New Ireland, and New Caledonia, Jama, Mallicolla, New Holland, and Van Dieman's Land. The more recent nation occupies Sumatra and the other islands of the Indian Sea, Otaheite and the Society Islands, the Friendly Islands, Morquesas, Ladrones, Marian and Caroline Islands, New Zealand, Sandwich, and Easter Island. The language of all the latter resembles the Malay, and there can be no doubt that they sprung from that race, and have spread by their ships over these distant spots. The black people are every where barbarous, and, according to Foster, have languages not agreeing with each other. In neither can we perceive any traces of the influence of climate. The latter race, scattered in the various parts of the vast island of New Holland, which has such variety of temperature, every where retains its black colour, although the climate at the English settlement is not much unlike that of England; and in Van Dieman's Land, extending to 45° south latitude, they are of a deep black, and have curled hair like the negroes.

Mr. Crawford, in his History of the Indian Archipelago, vol. ii. page 79, states that "the negro races who inhabit the mountains of the Malayan peninsula, in the lowest and most abject state of social existence, though numerically few, are divided into a great many different tribes, speaking as many different languages. Among the rude and scattered population of the island of Yima, it is believed that not less than forty different languages are spoken. On Jude and Flores we have also a multiplicity of languages; and among the cannibal population of Borneo it is not improbable that many hundreds are spoken. Civilization advances as we proceed westward; and in the considerable island of Sambawa there are but five tongues, in the civilised portion of Celebes not more than four, in the great island of Sumatra not above six, and in Java but two."

Both Mr. Crawford and Mr. Marsden, the author of the History of Sumatra, have traced what they call the great Polynesian language from Madagascar to New Guinea and the South-Sea Islands. Wherever the finer-formed and fairer race of people, with long hair, are found, this language may be traced. Mr. Crawford has given a specimen of the great Polynesian language, in which many of the words are exactly the same with those of the Church Missionary Society's New Zealand Grammar, and many more bear a close resemblance.

Mr. Marsden states "that, in the general character, particularly form and genius, of the innumerable languages spoken within the limits of the Indian islands, there is a remarkable resemblance, while all of them differ from those of any other nation of the world. This observation extends to every country from the north-west extremity of Sumatra to the western shores of New Guinea, and may be even carried to Madagascar to the west, the Phillipines to the east, and the remotest of Cook's discoveries to the south."

It is language and genius, and not shape or colour, that are the true tests of affinity of the different human races. Language, says Mr. Horne Tooke, cannot lie, and from the language of every nation we may with certainty collect its origin. If the nations of New Holland were of Malayan origin, some traces of this general language would be found among them; but, in the many hundred tongues that have been already found to exist in both this country and Van Dieman's Land, no one has been able to find the smallest vestige of it, or trace any affinity between their language and that of any other people. Each distinct community has its own peculiar language, and, at a distance of only a few miles, they cannot understand one another. This is even the case within the limits of the English colony.

It is a curious fact that Captain King, in his several voyages along the coast, and particularly in his visit to Endeavour River, could not verify a single word of Captain Cook's vocabulary, made there only fifty years ago. The word kangaroo, by which they thought, in the year 1770, the natives designated the animals so well known to us by that name, was found to mean nothing at all in the year 1820. Whether it is that the languages undergo a change in the course of a generation or two, or that different tribes occupy the same district of country at different periods, it is impossible to say; but the similarity between the natives of this country and the east insular negroes is too strong to admit a doubt of an identity of origin, whatever might have produced a difference in the hair, the only point in which they seem to me to be dissimilar.

The following history of the negro races of the Indian islands will, I suspect, equally apply to the natives of Australasia at no very distant period of time:—" The brown and negro race of the Archipelago may be considered to present, in their physical and moral character, a complete parallel with the white and negro races of the western world. The first have always displayed as eminent a relative superiority over the second, as the race of white men have over the negroes of the west: all the indigenous civilization of the Archipelago has sprung from them, and the negro race is constantly found in the most savage state. That race is to be traced from one extremity of the Archipelago to the other, but is necessarily less frequent where the most civilised race is most numerous, and seems entirely to have disappeared where the civilisation of the fairer race has proceeded farthest, as in Sumatra, Java, and perhaps Celebes; just as the Caribs and other savages of America have given way to the civilised invaders of Europe. The negro races of the Archipelago increase in number in the inverse ratio of improvement, or, in other words, as we proceed eastwards. In some of the Spice Islands their extirpation is matter of history. They are the principal races in some of the islands towards New Guinea, and nearly the sole inhabitants in the portion of that island itself which, from its physical character, we have a right to include within the Archipelago."* I do not by this mean to tax the English colonists with any intentional ill-treatment of their savage brethren. A few instances of cruelty towards them have occurred, and in Van Dieman's Land they have been persecuted in the most unfeeling and wanton manner: this has, however, been done by the lowest and worst description of the population, and contrary to the express orders of the government. Each of the governors, and the better class of settlers, have on all occasions cherished and protected them; but in spite of all their efforts they are still the same, and, notwithstanding the many benefits they derive from the colonists, it would appear there is something in civilised life which is inimical to them. Its very neighbourhood has an evident tendency to increase their degeneracy of character, and lessen their numbers.

